# Distinct Chemotypes of Putative *Commiphora gileadensis* (L.) C.Chr. Resins from Israel, Oman and Somalia

**DOI:** 10.3390/plants15162529

**Published:** 2026-08-21

**Authors:** Lumír Ondřej Hanuš

**Affiliations:** Institute for Drug Research, School of Pharmacy, Faculty of Medicine, Hebrew University, Ein Kerem Campus, Jerusalem 91120, Israel; lumirh@ekmd.huji.ac.il

**Keywords:** apharsemon, Balm of Gilead, *Commiphora gileadensis*, GC–MS, resin chemistry, linalyl acetate, chemotype, Somalia, Oman, Ein Gedi

## Abstract

*Commiphora gileadensis* (L.) C.Chr. has long been regarded as the most likely botanical source of the historical apharsemon (Balm of Gilead). Despite considerable historical interest, little is known about the chemical variability of geographically separated populations currently assigned to this taxon. In the present study, resin samples obtained from putative *C. gileadensis* accessions originating from Ein Gedi (Israel), Oman, and a Somalia-derived accession cultivated in Yotvata, Israel, were analyzed by GC–MS. In addition, freshly collected resin from the Somalia-derived accession was compared with the same resin sample stored under laboratory conditions for ten years. The Ein Gedi accession exhibited a monoterpene-rich profile dominated by sabinene (48.26%) and α-pinene (13.96%). In contrast, the Oman accession was characterized by copaene (17.94%), limonene (10.51%), β-elemene (8.06%), and isocembrol (6.31%). The Somalia-derived accession displayed a markedly different profile dominated by linalyl acetate (59.59%), accompanied by α-pinene (9.95%) and γ-terpinene (5.83%). After ten years of storage, linalyl acetate remained the dominant constituent (40.51%), and the characteristic pleasant aroma of the resin was largely preserved. The results demonstrate substantial phytochemical divergence among geographically separated accessions currently assigned to *C. gileadensis*. The Somalia-derived accession represents a distinct fragrance-rich chemotype whose unusual aroma profile and long-term stability may be of particular interest in future studies of balsam-producing *Commiphora* populations.

## 1. Introduction

The historical apharsemon, commonly identified with the Balm of Gilead, was one of the most highly valued aromatic and medicinal products of the ancient Near East. Classical authors such as Theophrastus, Strabo, Pliny the Elder, and Flavius Josephus described balsam cultivation in the regions of Jericho and Ein Gedi and emphasized the extraordinary economic value of the resin. In addition to its medicinal applications, apharsemon occupied an important role in perfumery, luxury trade, and religious traditions, contributing to its enduring reputation throughout antiquity and the medieval period [1,2].

Since the pioneering investigations of Pehr Forsskål [1] in Arabia during the eighteenth century, apharsemon has generally been associated with *Commiphora gileadensis* (L.) C.Chr. (syn. *Commiphora opobalsamum* (L.) Engl.). However, the extinction of the historical plantations of Judea and later Matarea in Egypt has prevented direct comparison between historical cultivated material and modern populations. Consequently, the relationship between present-day *C. gileadensis* populations and the celebrated balsam of antiquity remains incompletely understood [2,3].

Recent evidence has further complicated the interpretation of historical balsam-producing Commiphora populations. Sallon et al. [4] successfully germinated a nearly one-thousand-year-old Commiphora seed recovered from the Judean Desert and demonstrated, through molecular and phytochemical analyses, that the resulting plant represents a previously uncharacterized lineage within the genus. These findings indicate that historically important balsam-producing Commiphora populations may not correspond directly to extant populations currently assigned to *C. gileadensis* and highlight the need for integrated chemical and phylogenetic approaches when evaluating putative apharsemon material.

Members of the genus *Commiphora* are characterized by remarkable phytochemical diversity and are known to produce resins rich in mono-, sesqui-, and diterpenoids with important ecological, pharmacological, and aromatic functions. Comprehensive reviews have documented the occurrence of numerous volatile and non-volatile constituents in *C. gileadensis*, including monoterpenes, sesquiterpenes, triterpenes, flavonoids, and phenolic compounds. Considerable variation in chemical composition has been reported among plant parts, extraction procedures, and geographical sources.

Several phytochemical investigations of *Commiphora gileadensis* and *C. opobalsamum* have been published during the last two decades. Essential oils and extracts obtained from plants growing in Saudi Arabia and Israel have been shown to contain complex mixtures of mono-, sesqui-, and diterpenoids, often dominated by different groups of constituents depending on plant origin and experimental conditions. Al-Massarany et al. [5] reported detailed chemical characterization of *C. opobalsamum* essential oil, while Abbas et al. [6] investigated phytochemical constituents of Saudi material. More recently, Dudai et al. [7] examined the chemical composition and monoterpenoid enantiomeric distribution of apharsemon essential oils from Israel, further demonstrating the complexity of this species.

During the last decade, interest in *Commiphora gileadensis* has increased considerably. Several review papers have summarized current knowledge concerning its phytochemistry, traditional uses, biological activities, and pharmacological potential. These reviews highlight the occurrence of terpenoids, flavonoids, phenolic compounds, and other secondary metabolites, while also emphasizing the remarkable chemical variability reported among investigated plant materials [8,9,10,11]. At the same time, most published studies have focused on leaves, bark extracts, biological activities, or extraction methodologies, whereas comparative analyses of authentic resin samples from geographically distinct populations remain uncommon.

In particular, information regarding the chemical composition of balsam resins collected from different regions within the historical distribution range of *C. gileadensis* is surprisingly limited. While several studies have reported essential oil compositions from Saudi Arabian or Israeli material [5,6,7], few investigations have directly compared resin samples originating from geographically separated populations that may represent different chemotypes or lineages.

At the same time, recent reviews have emphasized the growing body of literature concerning the phytochemistry and biological activities of *C. gileadensis*, including antioxidant, antimicrobial, anti-inflammatory, and cytotoxic properties. Despite these advances, relatively little attention has been given to direct comparison of geographically distinct balsam-producing populations traditionally assigned to *C. gileadensis*. Consequently, the extent of chemical divergence among putative apharsemon populations from different parts of the historical Arabian–Horn of Africa distribution range remains poorly documented.

Particularly limited is information concerning resin chemistry and the preservation of fragrance during long-term storage. This issue is of special interest because historical sources consistently describe apharsemon not only as a medicinal substance but also as an exceptionally valuable perfume and aromatic material. The persistence of fragrance may therefore represent an important characteristic of historical balsam preparations, yet data concerning long-term chemical stability of authentic resin samples remain scarce.

In the present study, resin samples from three putative *Commiphora gileadensis* accessions were investigated by GC–MS. The accessions originated from Ein Gedi (Israel), the Sultanate of Oman, and a Somalia-derived accession cultivated in Yotvata, Israel. According to available records, the original Somali plant was selected in the wild because of its particularly pleasant fragrance and subsequently propagated vegetatively in Israel. In addition to comparing the three accessions, we examined the same Somalia-derived resin immediately after collection and again after ten years of storage under laboratory conditions.

The objectives of this work were to (i) compare the chemical composition of resin samples obtained from geographically distinct putative *C. gileadensis* accessions; (ii) evaluate the degree of phytochemical divergence among them; (iii) investigate chemical changes associated with prolonged storage of resin; and (iv) assess whether aroma-related characteristics may provide useful complementary information for future studies of balsam-producing *Commiphora* populations.

Recent work has demonstrated substantial habitat-related chemotypic variation within *Commiphora gileadensis*. Alsherif et al. [11] reported pronounced differences in secondary metabolite profiles between populations growing on basaltic and granitic substrates in Saudi Arabia, highlighting the considerable phytochemical plasticity of the species.

Chemotypic variation is increasingly recognized as an important source of intraspecific diversity in medicinal and aromatic plants. In many terpene-producing taxa, geographically separated populations differ not only in relative abundance of major constituents but may also exhibit distinct biosynthetic pathways, resulting in qualitatively different secondary metabolite profiles. Such variation can influence ecological interactions, fragrance characteristics, pharmacological properties, and commercial value. Consequently, characterization of chemotypes has become an important component of phytochemical and taxonomic investigations, particularly in genera containing economically valuable resin-producing species such as *Commiphora*. The identification of geographically structured chemical variation may therefore provide useful insights into both evolutionary differentiation and the historical utilization of balsam-producing plants.

## 2. Materials and Methods

### 2.1. Plant Material

Three putative *Commiphora gileadensis* (L.) C.Chr. accessions were investigated. The identities of the Ein Gedi and Somalia accessions were verified using flowering-stage specimens by Dr. Elaine Solowey and Ivan Čupič, respectively. Voucher specimens (HUJ-GED-2012-01 and HUJ-SOM-2011-01) were deposited at the School of Pharmacy. The Oman tree was identified in the field by a botanist with expertise in *Commiphora* taxonomy, who preferred to remain anonymous. A voucher specimen was deposited at the School of Pharmacy under accession number HUJ-OMA-2021-01.

#### 2.1.1. Ein Gedi Accession

Fresh resin was collected from plants cultivated in Ein Gedi, Israel, and identified as *Commiphora gileadensis*. The sample was analyzed on 15 January 2012.

#### 2.1.2. Oman Accession

Fresh resin originating from plants identified as *Commiphora gileadensis* growing in the Sultanate of Oman was analyzed on 6 September 2021.

#### 2.1.3. Somalia-Derived Accession

An accession cultivated in Yotvata, Israel, originated from a wild plant reportedly collected in Somalia by botanist Alon (James) Aharonson during the 1980s. According to available records, the original plant was selected because of its particularly pleasant aroma. Subsequently, the material was vegetatively propagated and cultivated in Israel. Dr. Elaine Solowey identified the plant as belonging to the genus *Commiphora* and considered *C. gileadensis* a possible assignment. Morphological similarities to the Ein Gedi accession were noted.

Fresh resin from this accession was analyzed on 28 December 2011 and re-analyzed after ten years of laboratory storage on 6 September 2021.

#### 2.1.4. Obtaining Resins

Resin samples were collected directly from the bark of the investigated trees. The Ein Gedi and Oman accessions yielded only small quantities of resin, amounting to several droplets in each case. In contrast, approximately 5 mL of resin was obtained from the Somalia-derived accession cultivated in Yotvata, Israel.

In all three accessions, the fresh resin was transparent, pale yellow to yellow, and possessed a viscosity comparable to that of liquid honey. The resins from the Ein Gedi and Oman accessions exhibited only a weak aroma. In contrast, the Somalia-derived resin was characterized by a pronounced, exceptionally pleasant fragrance, which represented one of the reasons why the original source plant had reportedly been selected in Somalia.

Part of the Somalia-derived resin collected in December 2011 was retained for long-term storage studies. The sample was stored under ordinary laboratory conditions in a transparent borosilicate glass vial fitted with a polyethylene cap and liner. After ten years of storage, the resin remained clear and light honey-yellow in appearance, with no visible signs of turbidity or darkening. Although its viscosity increased noticeably, the characteristic pleasant fragrance was largely preserved, consistent with the GC–MS results demonstrating retention of linalyl acetate as the dominant constituent.

Fresh resin samples were stored in airtight glass vials at 4 °C until analysis.

### 2.2. GC–MS Analysis

Prior to GC–MS analysis, resin samples were dissolved in *n*-hexane and filtered through a 0.45 μm nylon syringe filter (Filter Fix, 25 mm; SOLUFIX-SIMPLEPURE PTFE). The filtrates were adjusted to a final concentration of 0.1 mg mL^−1^ and analyzed without further derivatization.

Resin samples analyzed in 2021 were examined using an Agilent 7890B GC coupled to an Agilent 5977B MSD (Santa Clara, CA, USA) under the conditions described below. Historical chromatographic data obtained in 2011–2012 were generated using an HP G1800B GCD system equipped with an HP-5971 gas chromatograph (Palo Alto, CA, USA) and are included for comparison. Compound identification in all datasets was based on retention data and mass spectral matching.

Retention indices (RIs) were calculated relative to a homologous series of n-alkanes.

#### 2.2.1. Agilent GC–MS Conditions (2021 Analyses)

Chemical composition was determined by gas chromatography–mass spectrometry (GC–MS) using an Agilent 7890B gas chromatograph coupled with an Agilent 5977B mass selective detector and PAL 3 autosampler (RSI 85). Separation was achieved on an HP-5MS UI capillary column (30 m × 0.25 mm i.d., film thickness 0.25 μm; Agilent Technologies, Santa Clara, CA, USA). Helium was used as the carrier gas at a constant flow rate of 1.0 mL min^−1^. The oven temperature program was as follows: 35 °C for 5 min, increased to 150 °C at 5 °C min^−1^, and subsequently to 250 °C at 15 °C min^−1^, with a final hold of 90 min. The injector temperature was maintained at 250 °C and the detector temperature at 280 °C. Samples were injected in split mode (1:5). Mass spectra were acquired in EI mode (70 eV) over the mass range *m*/*z* 60–500.

#### 2.2.2. HP GC–MS Conditions (2011–2012 Analyses)

Chemical composition was determined by gas chromatography/mass spectrometry (GC/MS) in a Hewlett Packard G 1800B GCD system with an HP-5971 gas chromatograph with an electron ionization detector. The software used was GCD Plus ChemStation, (G1074B Version A.01.00) and the column was an Rtx^®^ 5MS low-bleed GC/MS column (30 m × 0.25 mm × 0.25 μm film thickness). For analysis, the column was kept at 50 °C for 4 min, and then the temperature was programmed from 50 °C to 280 °C at 8 °C/min; inlet 250 °C; detector 280 °C; splitless injection/purge time 1.0 min; initial temperature 100 °C; and initial time 4.0 min. The helium flow rate was 1 mL/min.

### 2.3. Compound Identification

Individual constituents were identified by comparison of retention times, retention indices, and mass spectra with authentic standards and reference libraries. Spectral matching was performed using the NIST/EPA/NIH Mass Spectral Library 2017, Wiley Registry of Mass Spectral Data (11th Edition), FFNSC3 library (2015), and Adams Essential Oil Library. Identification was further confirmed using commercially available reference standards, including α-pinene, camphene, β-pinene, myrcene, Δ3-carene, α-terpinene, *p*-cymene, limonene, 1,8-cineole, α-ocimene, trans-β-ocimene, γ-terpinene, terpinolene, linalool, geraniol, β-caryophyllene, α-humulene, nerolidol isomers, caryophyllene oxide, guaiol, and α-bisabolol, which were obtained from Restek (Bellefonte, PA, USA).

## 3. Results

### 3.1. Comparison of Commiphora gileadensis Accessions

The three investigated *Commiphora gileadensis* accessions exhibited markedly different chemical profiles despite their presumed taxonomic affinity (Table 1).

The Ein Gedi resin displayed a strongly monoterpene-dominated composition. Sabinene (48.26%) represented nearly half of the total identified constituents, followed by α-pinene (13.96%), *cis*-β-ocimene (7.37%), β-pinene (6.08%), and γ-terpinene (5.74%).

**Figure 1:**
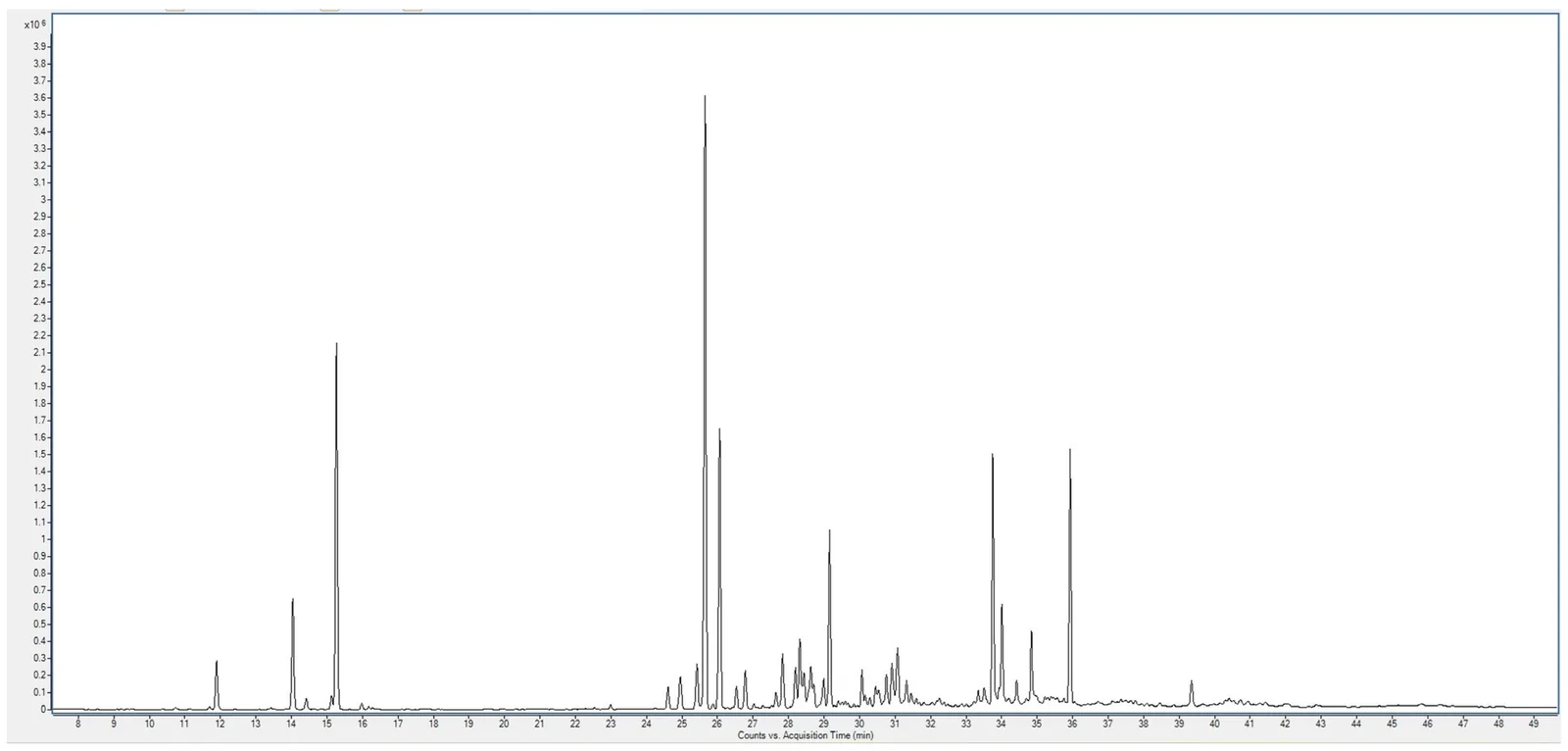
Representative GC-MS chromatogram of resin from the Oman accession of *Commiphora gileadensis*. Complete chromatograms of all analyzed samples are provided in the Supplementary Information (Figures S1–S4).

In contrast, the Oman accession (Figure 1) showed a substantially different profile characterized by copaene (17.94%), limonene (10.51%), β-elemene (8.06%), isocembrol (6.31%), and *m*-camphorene (5.92%). The resin therefore contained proportionally higher amounts of sesquiterpenes and diterpene-related compounds compared with the Ein Gedi accession.

The Somalia-derived accession cultivated in Yotvata differed strikingly from both previous samples. Its composition was dominated by linalyl acetate (59.59%), accompanied by α-pinene (9.95%) and γ-terpinene (5.83%). This exceptionally high concentration of linalyl acetate distinguished the sample from the Ein Gedi and Oman accessions and suggests the presence of a distinct chemotype within *C. gileadensis*.

### 3.2. Effect of Storage on the Somalia-Derived Accession

Comparison of fresh resin and the same resin after ten years of laboratory storage revealed substantial compositional changes (Table 2). Several monoterpene hydrocarbons decreased markedly during storage. For example, α-pinene decreased from 9.95% to 1.69%, while γ-terpinene declined from 5.83% to 0.03%.

Despite these changes, linalyl acetate remained the dominant constituent, decreasing from 59.59% in the fresh resin to 40.51% after storage. At the same time, isocembrol increased from 0.52% to 1.86%, and several oxygenated compounds that were absent or present only in trace amounts in the fresh resin became detectable in the aged sample. These changes are consistent with volatilization of highly volatile monoterpene hydrocarbons together with gradual oxidation and chemical transformation processes occurring during long-term storage.

Nevertheless, the overall chemical profile remained clearly recognizable after ten years, indicating a remarkable preservation of the characteristic aromatic composition. Linalyl acetate continued to be the predominant constituent of the aged resin.

## 4. Discussion

### 4.1. Evidence for Distinct Chemotypes

The observed differences should be interpreted cautiously because the historical and recent samples were analyzed using different GC-MS instruments and chromatographic methods.

Nevertheless, the dominant constituents and overall chemotypic patterns were sufficiently distinct to allow meaningful comparison.

The three accessions investigated in this study exhibited strikingly different chemical profiles. The Ein Gedi resin was dominated by sabinene, the Oman accession by sesquiterpenes and diterpenoids, and the Somalia-derived accession by linalyl acetate. Such differences suggest the presence of distinct chemotypes within materials currently assigned to *Commiphora gileadensis*.

The present findings are consistent with recent evidence indicating substantial chemotypic variation within *Commiphora gileadensis*. Alsherif et al. [12] demonstrated pronounced habitat-specific differences in the secondary metabolite profiles of *C. gileadensis* populations growing on basaltic and granitic substrates in Saudi Arabia. These authors identified distinct chemotypes characterized primarily by sesquiterpenes and oxygenated sesquiterpenes and concluded that edaphic factors may significantly influence secondary metabolite production.

The chemotypes observed in the present study differ substantially from those reported previously for Saudi Arabian populations of *C. gileadensis*. Alsherif et al. [12] described habitat-dependent chemotypes dominated primarily by sesquiterpenes and oxygenated sesquiterpenes, whereas the Somalia-derived accession investigated here was characterized by an exceptionally high abundance of linalyl acetate (59.6%). Similarly, the sabinene-rich profile observed in the Ein Gedi accession has not been prominently reported in recent studies. These observations suggest that the chemotypic diversity within putative *C. gileadensis* may be broader than currently recognized.

Further evidence of the remarkable metabolic plasticity of *C. gileadensis* was provided by Kutby et al. [13], who compared wild and cultivated plants and observed substantial shifts in secondary metabolite composition. Cultivated plants exhibited reduced chemical richness but increased abundance of selected bioactive sesquiterpenoids, particularly viridiflorol, demonstrating that dominant constituents can vary considerably even within a relatively limited geographic context. These findings suggest that *C. gileadensis* possesses a highly flexible secondary metabolism capable of generating markedly different chemical profiles under differing environmental and cultivation conditions.

In addition to environmental influences, geographic isolation and subsequent genetic drift may also contribute to the observed chemotypic divergence. Long-term separation of populations may promote both genetic differentiation and divergence of secondary metabolite biosynthetic pathways. This possibility is particularly relevant in the case of *C. gileadensis*, whose present distribution includes geographically isolated populations across Arabia and northeastern Africa. This hypothesis is supported by recent molecular investigations described below.

Additional evidence for differentiation among geographically separated populations of *C. gileadensis* has been provided by recent chloroplast genomic studies. Al-Andal [14] compared chloroplast genomes of *C. gileadensis* from Saudi Arabia and Oman and identified extensive genetic divergence, including approximately 1200 sequence variations, numerous SNPs and indels, and differences in chloroplast gene content. The authors concluded that populations from the two regions have undergone distinct evolutionary trajectories. Such genetic differentiation provides a plausible framework for interpreting the pronounced phytochemical divergence observed among the resin samples investigated in the present study and suggests that at least part of the observed chemotypic variation may reflect underlying genetic structure rather than solely environmental influences.

The magnitude of the chemical differences observed among the investigated accessions raises the possibility that some populations currently assigned to *C. gileadensis* could represent genetically differentiated lineages or even taxonomically unresolved entities. Although the present data do not allow taxonomic conclusions, the combination of marked chemotypic divergence and recently reported chloroplast genome differentiation [14] highlights the need for integrated molecular and phytochemical studies.

Although the present dataset is limited in size, the magnitude of the observed differences exceeds what might be expected from normal quantitative variation within a single chemotype. The dominant constituent of the Somalia-derived accession, linalyl acetate, accounted for nearly 60% of the detected volatile fraction, whereas the major compounds of the Ein Gedi and Oman accessions belonged to entirely different terpene classes. Such pronounced shifts in dominant metabolites are often indicative of substantial differences in underlying biosynthetic pathways and regulatory mechanisms.

Similar patterns have been reported in other aromatic plant groups where genetically differentiated populations produce distinct terpene assemblages despite taxonomic assignment to the same species. Therefore, the results support the possibility that the investigated accessions represent chemically differentiated lineages deserving further comparative study.

However, the differences observed in the present study appear even more pronounced. Not only were the investigated accessions separated geographically across Israel, Oman, and Somalia-derived material cultivated in Israel, but their dominant constituents also differed fundamentally. Particularly noteworthy was the Somalia-derived accession, whose resin contained 59.6% linalyl acetate, a profile not reported among the Saudi Arabian chemotypes described by Alsherif et al. [12], where sesquiterpenes and oxygenated sesquiterpenes predominated.

### 4.2. The Somalia-Derived Fragrance-Rich Chemotype

The most notable finding was the unique chemistry of the Somalia-derived accession.

Unlike the other samples, its resin was dominated by linalyl acetate, a compound strongly associated with floral and perfume-like aromas.

According to available records, the original plant was selected in Somalia because of its particularly pleasant fragrance. The chemical data obtained in the present study support this observation. The extraordinarily high concentration of linalyl acetate distinguishes this accession from both the Ein Gedi and Oman samples.

Linalyl acetate is widely recognized as one of the principal contributors to the floral fragrance of lavender oils and is frequently associated with desirable perfumery properties. Its unusually high abundance in the Somalia-derived accession therefore distinguishes this resin not only from the other accessions examined here but also from most published reports concerning *C. gileadensis* chemistry [7,8,9,10,11].

From an organoleptic perspective, the dominance of linalyl acetate is particularly noteworthy. This compound is widely recognized as one of the principal contributors to the pleasant floral aroma of lavender and several other perfume plants. The occurrence of such a high concentration within a *Commiphora* resin is unusual and may explain the long-standing observation that the original Somali source plant possessed an exceptionally attractive fragrance. Although sensory evaluation was not performed systematically in the present study, the chemical composition provides an objective basis for the distinctive aromatic qualities noted during cultivation and handling of the material.

### 4.3. Aroma Persistence During Prolonged Storage

Equally noteworthy is the preservation of aroma following ten years of storage. Although substantial decreases occurred in several monoterpenes, linalyl acetate remained the dominant constituent, and the resin retained its characteristic pleasant fragrance. Similar storage-related changes have been reported in terpene-rich essential oils, where losses of volatile monoterpene hydrocarbons and the formation of oxygenated derivatives are commonly attributed to evaporation and oxidative transformation processes during storage [15,16].

The observed stability may have broader implications for understanding the historical value of balsam resins. Highly prized aromatic products were frequently transported over considerable distances and often stored for extended periods before use. A resin capable of maintaining a recognizable fragrance profile despite partial volatilization and oxidation of monoterpenes would possess practical advantages in trade and perfumery. Although the present findings cannot be extrapolated directly to historical apharsemon preparations, they demonstrate that resin-based aromatic systems may retain their characteristic olfactory identity for remarkably long periods under ordinary storage conditions.

This observation may be relevant to historical descriptions of apharsemon as an exceptionally valued perfume and aromatic substance. While no direct connection between the Somalia-derived accession and the historical Balm of Gilead can be established, aromatic quality and long-term fragrance stability may represent important but previously underappreciated traits in evaluations of balsam-producing *Commiphora* populations.

### 4.4. Implications for Apharsemon Research

The present findings support the view that populations currently assigned to *Commiphora gileadensis* constitute a chemically heterogeneous complex. Whether this variability reflects environmental influences, geographical differentiation, chemotypic variation, or taxonomic complexity remains uncertain. Recent molecular evidence further emphasizes this uncertainty. Sallon et al. [4] demonstrated that a nearly millennium-old *Commiphora* seed recovered from the Judean Desert belonged to a previously uncharacterized lineage within the genus rather than to any currently sampled population. Their findings indicate that historically significant balsam-producing *Commiphora* lineages may not be adequately represented by modern material and support the need for integrated phytochemical, morphological, and molecular investigations of putative apharsemon populations. Future studies integrating phytochemistry, morphology, archaeobotany, and molecular systematics will be required to resolve these questions and to clarify the relationship between modern *Commiphora* populations and the historical source of apharsemon.

A limitation of the present study is the absence of biological replicates and the use of different GC–MS systems for historical and recent analyses. Consequently, the results should be interpreted primarily as evidence of chemotypic differences rather than precise quantitative comparisons among populations.

### 4.5. Limitations and Future Directions

Several limitations of the present study should be acknowledged. The investigation was based on a small number of resin samples and did not include biological replication within each accession. Furthermore, historical and recent samples were analyzed using different GC-MS instruments and chromatographic programs, which may influence the detection of minor constituents. Nevertheless, the major compounds responsible for the observed chemotypic patterns were sufficiently abundant to permit meaningful comparison. Future studies should include larger sampling across the Arabian Peninsula and the Horn of Africa, together with molecular identification, morphometric characterization, and standardized analytical protocols. Integration of phytochemical and genomic approaches will be essential for clarifying relationships among modern *Commiphora* populations and for assessing their possible relevance to historically important balsam-producing lineages.

## 5. Conclusions

The present study demonstrates substantial phytochemical divergence among three geographically distinct accessions currently assigned to *Commiphora gileadensis*. Resin from the Ein Gedi accession was characterized by a sabinene-dominated monoterpene profile, whereas the Oman accession exhibited a markedly different composition rich in sesquiterpenes and diterpenoid-related constituents. In contrast, the Somalia-derived accession cultivated in Israel represented a unique fragrance-rich chemotype dominated by linalyl acetate. The magnitude of these compositional differences suggests the existence of pronounced chemotypic differentiation within material currently referred to as *C. gileadensis*.

Comparison of fresh and ten-year-old resin from the Somalia-derived accession further revealed substantial changes in the abundance of several volatile monoterpenes, likely resulting from volatilization together with gradual oxidative and transformation processes during storage. Despite these changes, linalyl acetate remained the dominant constituent, and the resin retained its characteristic pleasant fragrance, indicating remarkable long-term preservation of its aromatic identity.

Although the limited number of samples and the use of different analytical systems require cautious interpretation, the results highlight the considerable chemical heterogeneity of putative *C. gileadensis* populations. Future investigations integrating phytochemical, morphological, and molecular approaches across a broader geographic range will be necessary to clarify the relationships among these populations and to better understand their relevance to the historical balsam-producing *Commiphora* traditionally associated with apharsemon (Balm of Gilead).

## Figures and Tables

**Table 1:** Chemical composition (%) of *Commiphora gileadensis* resin samples from Ein Gedi, Oman, and the Somalia-derived (Yotvata) accession.

Compound	Ein Gedi (%)	Oman (%)	Yotvata Fresh (%)	RI
2,4-Dimethylheptane	nd	0.01	nd	821
Nonane	nd	0.05	nd	900
α-Thujene	nd	0.08	1.15	929
α-Pinene	13.96	1.41	9.95	937–939
Camphene	nd	nd	1.19	952
Sabinene	48.26	0.01	2.82	974–976
β-Pinene	6.08	0.05	1.18	979–980
β-Myrcene	4.28	3.11	2.29	991
α-Phellandrene	0.65	0.35	0.19	1005
Δ3-Carene	nd	0.02	nd	1011
α-Terpinene	1.86	nd	nd	1018
*p*-Cymene	0.32	0.39	0.99	1025–1026
Limonene	nd	10.51	1.60	1030
β-Phellandrene	3.10	nd	nd	1031
1,8-Cineole	nd	nd	1.15	1032
*cis*-β-Ocimene	7.37	nd	0.35	1040
*trans*-β-Ocimene	0.09	nd	0.62	1049–1050
γ-Terpinene	5.74	nd	5.83	1060–1062
*trans*-Sabinene hydrate	1.12	nd	nd	1068
Terpinolene	0.19	nd	0.78	1088
Linalool	0.41	nd	0.26	1098–1099
Alloocimene	0.93	nd	nd	1129
Terpinen-4-ol	0.42	nd	nd	1175–1177
α-Terpineol	0.67	nd	nd	1189
*cis*-Sabinene hydrate acetate	0.07	nd	nd	1219
Linalyl acetate	nd	nd	59.59	1257
Bornyl acetate	2.56	nd	0.31	1285
α-Terpinyl acetate	nd	nd	2.20	1350
α-Longipinene	nd	1.14	nd	1353
Neryl acetate	nd	nd	0.35	1364
Cyclosativene	nd	1.38	nd	1368
Ylangene	nd	0.08	nd	1372
Copaene	nd	17.94	nd	1376
Geranyl acetate	nd	nd	0.67	1382
β-Elemene	nd	8.06	nd	1391
α-Gurjunene	nd	0.65	nd	1409
β-Caryophyllene	nd	1.15	0.23	1419
γ-Elemene	nd	0.04	nd	1434
α-Bergamotene	nd	nd	0.46	1435
Aromadendrene	nd	0.10	nd	1440
β-Farnesene	nd	nd	0.03	1443
Guaia-6,9-diene	nd	0.10	nd	1444
Humulene	nd	0.46	nd	1454
Alloaromadendrene	nd	1.67	nd	1461
γ-Muurolene	nd	1.31	nd	1477
Germacrene D	0.35	1.97	1.69	1480–1481
β-Selinene	nd	1.38	nd	1486
α-Selinene	nd	1.26	nd	1492
Bicyclogermacrene	nd	nd	0.08	1494
α-Muurolene	nd	0.69	nd	1499
γ-Cadinene	nd	0.78	nd	1513
δ-Cadinene	nd	4.77	nd	1524
α-Cadinene	nd	0.19	nd	1538
α-Calacorene	nd	0.15	nd	1542
Elemol	nd	0.15	nd	1549
Spathulenol	nd	0.94	nd	1576
Copaborneol	nd	0.57	nd	1592
τ-Cadinol	nd	1.53	nd	1640
β-Eudesmol	nd	1.53	nd	1649
α-Cadinol	nd	0.55	nd	1653
(+)-Jatamansone	nd	0.14	nd	1677
Cembrene	nd	nd	0.64	1939
Neocembrene A	nd	nd	0.35	1959
m-Camphorene	nd	5.92	nd	1960
p-Camphorene	nd	2.45	nd	1995
Cembrene C	nd	nd	0.14	2020
Isocembrol	nd	6.31	0.52	2073
Hexadecanoic acid ethyl ester	0.05	nd	nd	2144
9-Octadecenoic acid ethyl ester	0.41	nd	nd	2179
Octadecanoic acid ethyl ester	0.02	nd	nd	2194
Squalene	1.10	nd	nd	2790
Total identified (%)	98.91	82.00	97.61	

nd = not detected.

**Table 2:** Chemical composition (%) of fresh and 10-year-old resin from the Somalia-derived (Yotvata) accession of *Commiphora gileadensis*.

Compound	Fresh Resin %	10-Year-Old Resin %	RI
tricyclene		0.01	925
α-thujene	1.15	0.10	929
α-pinene	9.95	1.69	937
camphene	1.19	0.29	952
sabinene	2.82	0.21	974
β-pinene	1.18	0.18	979
6-methyl-5-hepten-2-one	nd	0.02	986
β-myrcene	2.29	0.75	991
Δ^2^-carene	nd	0.02	1001
α-phellandrene	0.19	nd	1005
*p*-cymene	0.99	1.05	1025
limonene	1.60	0.17	1030
1,8-cineole	1.15	0.06	1032
*cis*-β-ocimene	0.35	0.20	1040
*trans*-β-ocimene	0.62	0.44	1049
γ-terpinene	5.83	0.03	1060
sabinene hydrate	nd	0.03	1066
*cis*-linalool oxide	nd	0.10	1074
*trans*-linalool oxide	nd	0.11	1086
terpinolene	0.78	nd	1088
6,7-epoxymyrcene	nd	0.03	1090
linalool	0.26	0.81	1099
α-campholenal	nd	0.02	1125
*neo*-*allo*-ocimene	nd	0.06	1131
*trans*-pinocarveol	nd	0.03	1139
*trans*-verbenol	nd	0.10	1144
borneol	nd	0.02	1166
terpinen-4-ol	nd	0.03	1177
*p*-cymen-8-ol	nd	0.09	1183
α-terpineol	nd	0.03	1189
myrtenol	nd	0.04	1195
1,2-benzisothiazole	nd	0.03	1221
carvacrol methyl ether	nd	0.02	1244
linalyl acetate	59.59	40.51	1257
β-terpinyl acetate	nd	0.02	1272
bornyl acetate	0.31	0.28	1285
α-terpinyl acetate	2.20	1.92	1350
neryl acetate	0.35	0.24	1364
geranyl acetate	0.67	0.42	1382
*cis*-α-bergamotene	nd	0.34	1415
β-caryophyllene	0.23	nd	1419
α-bergamotene	0.46	nd	1435
β-farnesene	0.03	nd	1443
germacrene D	1.69	nd	1481
bicyclogermacrene	0.08	nd	1494
spathulenol	nd	0.40	1576
cembrene	0.64	0.90	1939
neocembrene A	0.35	nd	1959
cembrene C	0.14	nd	2020
isocembrol	0.52	1.86	2073
cembrenol	nd	1.74	2162
	Σ = 97.61	Σ = 55.40	

nd = not detected.

## Data Availability

The data presented in this study are available from the corresponding author upon reasonable request.

## Supplementary Materials

The following supporting information can be downloaded at https://www.mdpi.com/article/10.3390/plants15162529/s1, Figure S1: GC-MS chromatogram of fresh resin from the Somalia-derived accession cultivated in Yotvata, Israel; Figure S2: GC-MS chromatogram of resin from the Somalia-derived accession cultivated in Yotvata, Israel, re-analyzed after ten years of laboratory storage.; Figure S3: GC-MS chromatogram of fresh resin from the Ein Gedi accession, Israel; Figure S4: GC-MS chromatogram of fresh resin from the Oman accession.
