# Supplementary material for: Distinct Chemotypes of Putative Commiphora gileadensis (L.) C.Chr. Resins from Israel, Oman and Somalia
Source: Plants (Basel). 2026 Aug 21;15(16):2529. doi: 10.3390/plants15162529 (PMC13517040; doi:10.3390/plants15162529)
Supplement: Supplementary file 1 [file plants-15-02529-s001.zip › plants-4492989-supplementary.pdf]

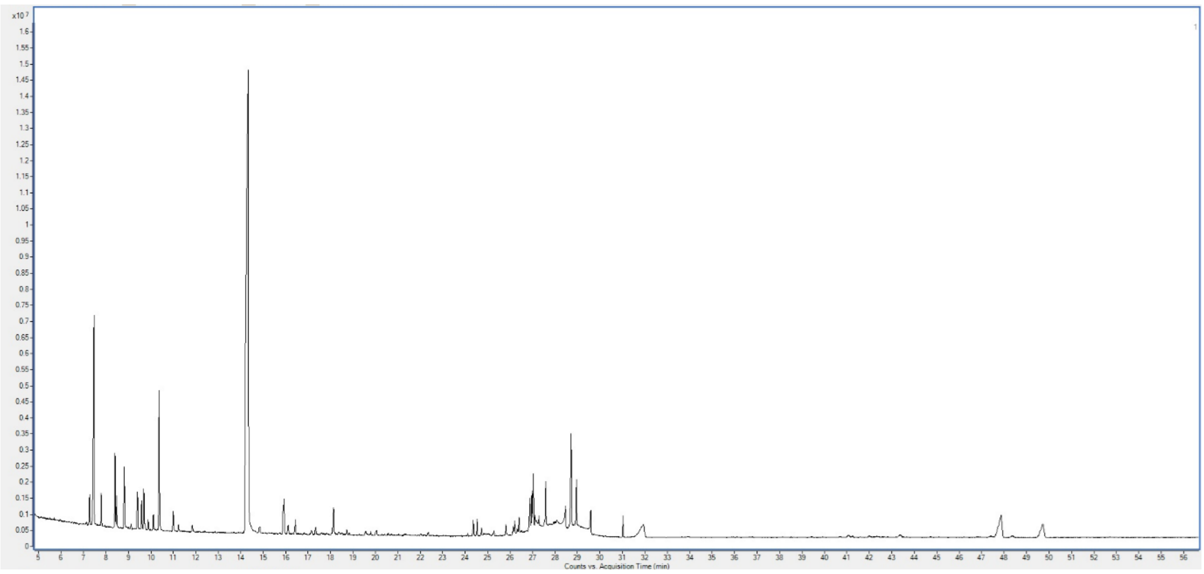

Figure S1. GC-MS chromatogram of fresh resin from the Somalia-derived accession cultivated in Yotvata, Israel.

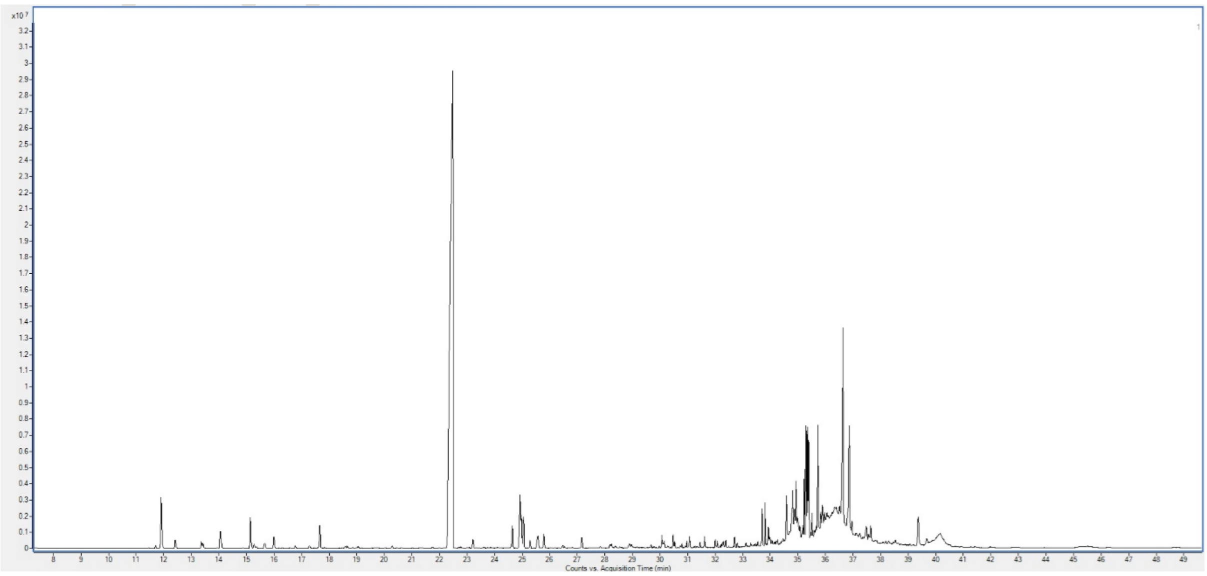

Figure S2. GC-MS chromatogram of resin from the Somalia-derived accession cultivated in Yotvata, Israel, re-analyzed after ten years of laboratory storage.

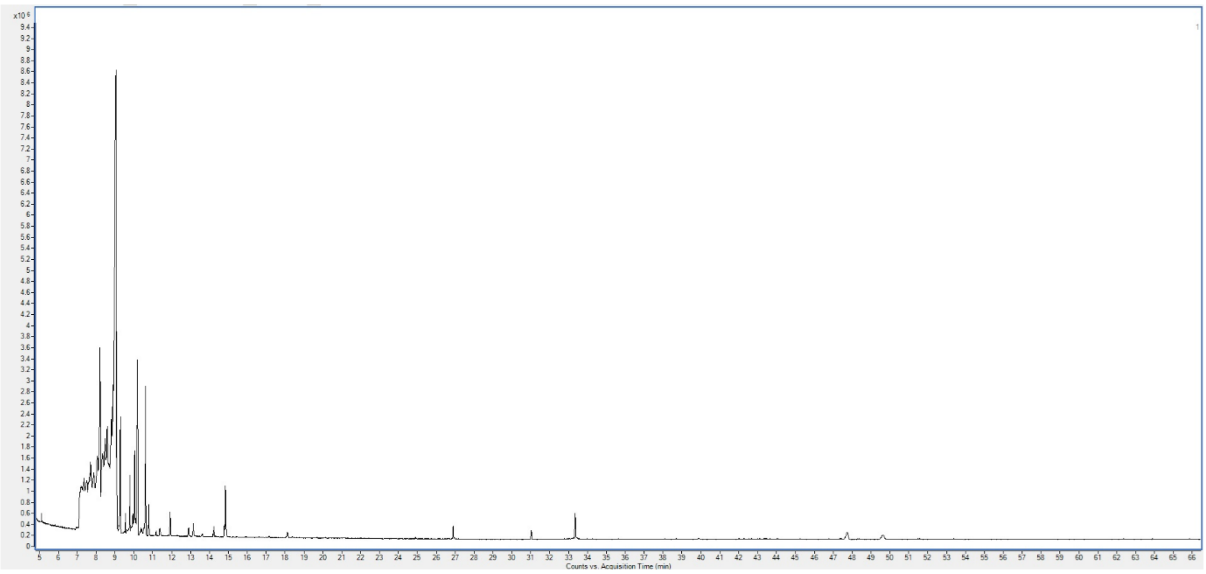

Figure S3. GC-MS chromatogram of fresh resin from the Ein Gedi accession, Israel.

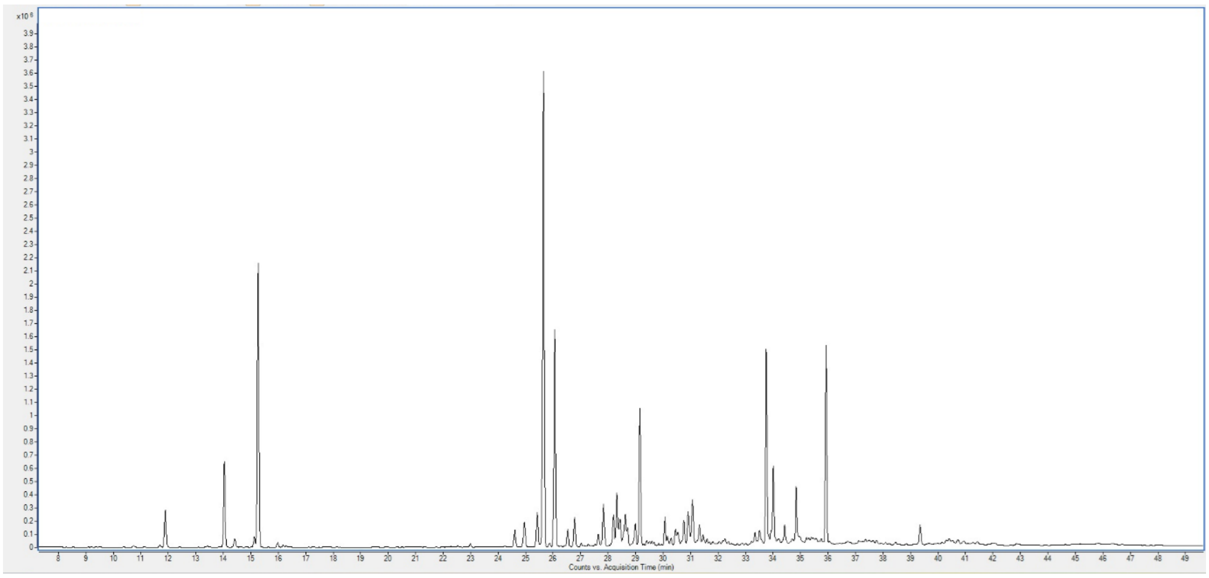

Figure S4. GC-MS chromatogram of fresh resin from the Oman accession.
